# Effects of radiation mitigating amino acid mixture on mice of different sexes

**DOI:** 10.3389/fpubh.2024.1394023

**Published:** 2024-06-03

**Authors:** Mang Xiao, Lisa Hull, Alex Zizzo, Bin Lin, Min Zhai, Li Wang, Wanchang Cui

**Affiliations:** ^1^Radiation Countermeasures Program, Armed Forces Radiobiology Research Institute, Uniformed Services University of the Health Sciences, Bethesda, MD, United States; ^2^Henry M Jackson Foundation for the Advancement of Military Medicine, Inc., Bethesda, MD, United States

**Keywords:** radiation injury, medical countermeasures, male and female mice, cytokine, mitigation, amino acids, survival

## Abstract

To date, few FDA-approved medical countermeasures are available for addressing hematopoietic acute radiation syndrome (H-ARS). In this study, we present our latest research findings focusing on the evaluation of a novel radiation mitigator known as the mitigating amino acid mixture (MAAM). MAAM is composed of five amino acids as the recently reported amino acid-based oral rehydration solution for mitigating gastrointestinal (GI)-ARS. CD2F1 male and female mice were exposed to ^60^Co-γ total body irradiation (TBI) at 9.0 or 9.5 Gy. Following irradiation, mice were orally administered with MAAM or a saline vehicle control once daily for a duration of 14 days, commencing 24 h after TBI. Mouse survival and body weight change were monitored for 30 days after irradiation. Complete blood counts (CBCs), bone marrow (BM) stem and progenitor cell survival (clonogenicity), and a serum cytokine antibody array were analyzed using samples from day 30 surviving mice. Our data revealed that MAAM treatment significantly enhanced survival rates in irradiated male CD2F1 mice, and the survival rate increased from 25% in the vehicle control group to 60% in the MAAM-treated group (*p* < 0.05) after 9.0 Gy TBI. The number of BM colonies significantly increased from 41.8 ± 6.4 /10^4^ cells (in the vehicle group) to 78.5 ± 17.0 /10^4^ cells (in the MAAM group) following 9.0 Gy TBI. Furthermore, MAAM treatment led to a decrease in the levels of six cytokines/proteins [cluster of differentiation 40 (CD40), interleukin (IL)-17A, C–X–C motif chemokine 10 (CXCL10/CRG-2), cutaneous T cell-attracting chemokine (CTACK), macrophage inflammatory protein (MIP)-3β, and IL-1β] and an increase in the levels of five other cytokines/proteins [IL-3Rβ, IL-5, leptin, IL-6, and stem cell factor (SCF)] in mouse serum compared to the vehicle group after 9.0 Gy TBI. However, similar alleviating effects of MAAM were not observed in the irradiated CD2F1 female mice. The serum cytokine profile in the irradiated female mice was different compared to the irradiated male mice. In summary, our data suggest that the beneficial effects of the mitigative amino acid combination treatment after radiation exposure may depend on sex.

## Introduction

Radiation exposures from medical, accidental, or terrorist radiation/nuclear incidents have the potential to affect the function of many biological systems and result in multiple organ injuries and health problems, depending on the type, dose, and dose rate of radiation. Recently, six medical countermeasures (MCMs), namely, Neupogen, Neulasta, Leukine, Nplate, and two biosimilar medicines, Neulasta Stimufend (peg-fpgk) and Udenyca (peg-cbqv), have been approved by the U.S. Food and Drug Administration (FDA) to treat hematopoietic complications following acute radiation exposure (1). However, MCMs to mitigate/treat radiation-induced other organ injuries, such as gastrointestinal (GI) and neural injuries, are not yet available.

Radiation serves as a nearly universal stressor for all living organisms. Therefore, it is presumed that conserved mechanisms exist to address radiation-induced stress, from single-cell organisms to humans. Recently, we have identified that specific amino acid permeases (membrane proteins involved in the transport of amino acids into cells) played important roles in determining *Cryptococcus neoformans*’ radiation sensitivity (2). Furthermore, a series of studies reported that treatment with a specific amino acid-based oral rehydration solution (AA-ORS) decreased intestinal paracellular permeability and plasma endotoxin, increased electrolyte absorption, and improved intestinal mucosal barrier function in male Swiss mice exposed to total body irradiation (TBI) (3–5). Oral drug administration represents a favorable option for the development of radiation countermeasures, particularly in the context of radiation casualties. Furthermore, the ingredients in the AA-ORS are all listed as “Generally Recognized as Safe” by the US FDA, exempting them from standard Federal Food, Drug, and Cosmetic Act (FFDCA) requirements. Therefore, specific amino acid mixtures have the potential to modify radiation responses.

An increasing number of reports have demonstrated that the same type and dose of radiation exposure could result in different levels of injury in male and female mice (6–9); therefore, the same MCM candidates may have different efficacy in different sexes of animal models (10), indicating the complex mechanisms of radiation injury. There is a pressing need for new MCMs that demonstrate high efficacy and do not pose any toxicity to effectively mitigate and treat acute radiation syndrome (ARS) in different sexes of radiation victims. Therefore, we conducted tests to assess the alleviating effects of mitigating amino acid mixture (MAAM) on the survival of mice following exposure to lethal doses of TBI in both male and female CD2F1 mice. This MAAM includes the same five amino acids and electrolytes previously reported by Gupta et al. (3). Our investigation focused on the potential of MAAM as a radiation countermeasure in both male and female mice.

## Materials and methods

### Ethics statement

Animals were housed in an Association for Assessment and Accreditation of Laboratory Animal.

Care (AAALAC)-approved facility at the Uniformed Services University of the Health Sciences (USUHS). All animal study procedures, including housing, drug administration, irradiation, survival study, and blood and tissue collection, were reviewed and approved by the USUHS Institutional Animal Care and Use Committee (IACUC), and all experiments were performed in accordance with the USUHS-IACUC and the USUHS Department of Laboratory Animal Resources (DLAR) relevant guidelines and regulations.

### Mice and animal care

CD2F1 male and female mice (ENVIGO, Indianapolis, IN) were used according to the methods previously described (11). Twelve- to 14-week-old mice with an average weight of 20–25 g for female mice and 25–30 g for male mice at the time of irradiation were chosen randomly for each experimental group and housed in an AAALAC-approved facility at the USUHS. Animal rooms were maintained at 20–26°C with 30–70% humidity on a 12 h light/dark cycle. Commercial rodent chow (Harlan Teklad Rodent Diet 8,604) was available *ad libitum,* as was acidified water (pH = 2.5–3.0) to control opportunistic infections.

### Irradiation and drug

Male and female mice received TBI in a bilateral radiation field at AFRRI’s ^60^Co-γ facility. The alanine/electron spin resonance (ESR) dosimetry system (American Society for Testing and Materials, Standard E 1607) was used to measure dose rates (to water) in the cores of acrylic mouse phantoms. The midline tissue dose to the male mice was 9.0 Gy, and to the female mice was 9.0 Gy or 9.5 Gy at a dose rate of 0.6 Gy/min. The day of irradiation was considered day 0.

The composition of the MAAM used in this study is listed in Table 1. MAAM or saline as vehicle control (200 μl/mouse), was administered through oral gavage once daily for 14 days, starting at 24-h post-TBI. The five amino acids are L-threonine, L-valine, L-serine, L-tyrosine, and L-aspartic acid. Among them, L-threonine and L-valine are essential amino acids. The electrolyte solution was composed of sodium citrate tribasic dihydrate, potassium chloride, calcium chloride dihydrate, magnesium chloride hexahydrate, and sodium chloride. All chemicals were purchased from Sigma (MilliporeSigma, Rockville, MD). The final solution was adjusted to pH 4.2 and filtered with 0.22 μm filters. The osmolarity of MAAM is 167.1 mOsm/L.

**Table 1:** Composition of the mitigating amino acid mixture (MAAM).

**Name**	**Sigma catalog #**	**Linear formula**	**CAS No.**	**Molecular weight (Da)**	**Molarity (mmol/L)**	**Concentration (g/L)**
L-threonine	T8625	CH_3_CH(OH)CH(NH_2_)CO_2_H	72-19-5	119.12	8.4	1
L-valine	V0500	(CH_3_)_2_CHCH(NH_2_)CO_2_H	72-18-4	117.15	10.2	1.2
L-serine	S4500	HOCH_2_CH(NH_2_)CO_2_H	56-45-1	105.09	10.5	1.1
L-tyrosine	T3754	4(HO)C_6_H_4_CH_2_CH(NH_2_)CO_2_H	60-18-4	181.19	1.1	0.2
L-aspartic acid	A9256	HO_2_CCH_2_CH(NH_2_)CO_2_H	56-84-8	133.10	8.26	1.1
Sodium citrate tribasic dihydrate	S4641	HOC(COONa)(CH_2_COONa)_2_ · 2H_2_O	6132-04-3	294.10	3.35	0.985
Potassium chloride	P5405	KCl	7447-40-7	74.55	10	0.746
Calcium chloride dihydrate	C7902	CaCl_2**·**_ 2H_2_O	10035-04-8	147.01	1.2	0.176
Magnesium chloride hexahydrate	M2393	MgCl_2_ · 6H_2_O	7791-18-6	203.30	1.2	0.244
Sodium chloride	S5886	NaCl	7647-14-5	58.44	44	2.571

pH 4.2; osmolarity 167.1 mOsm/L; Da, Dalton.

### Survival for 30 days and body weight measurement

After TBI and MAAM or vehicle control treatment, mice were closely monitored for 30 days by the researchers in addition to the regular health check-ups by vivarium staff (*N* = 20/group of mice). During the 30-day period, the USUHS-IACUC Policy-20 (Establishment of early endpoints in a mouse TBI model) was followed (12). Morbid animals were examined at least three times daily, and the moribund animals considered to have arrived at the endpoint were euthanized by CO_2_ inhalation plus confirmatory cervical dislocation. The basal body weight was measured 1 day before irradiation (day −1). Furthermore, body weight was monitored on days 7, 14, 21, and 28 post-irradiations.

### Mouse complete blood cell counts and serum and tissue preparation

All methods were developed in our group as previously described (13–15). Mice were humanely euthanized for whole blood, serum, and tissue collection. Euthanasia was carried out in accordance with the recommendations and guidelines of the American Veterinary Medical Association. Mice were deeply anesthetized prior to collecting whole blood through a cardiac blood draw. Confirmatory cervical dislocation was performed while the animal was still anesthetized in accordance with the approved IACUC protocol. The blood was immediately divided into two tubes. The samples in EDTA tubes were used for peripheral blood cell counts using a clinical hematology analyzer (Element HT5, Heska Co. Loveland, CO), and samples in the BD Microtainer Gold tubes were left undisturbed on racks. Following a 30 min coagulation at room temperature, the sera were well separated from the gel by a 10 min centrifugation in an Eppendorf centrifuge at 10,000 rpm. The sera were collected and stored at −80°C for future studies. Once blood collection from an individual mouse and mouse euthanasia were completed, the mouse femurs and humeri were collected.

### Clonogenicity assay

Bone marrow (BM) cells were collected from mouse femurs and humeri. After erythrocytes were lysed with erythrocyte lysis buffer (Qiagen GmbH, Hilden, Germany), total BM myeloid cell viability from each mouse was measured using Trypan blue staining. Clonogenicity of mouse BM cells was quantified in standard semisolid cultures in triplicates using 1 mL of MethoCult GF+ system (including SCF, IL-3, IL-6, and erythropoietin) for mouse cells (Cat # 03444, STEMCELL Technologies) according to the manufacturer’s instructions, as previously described (16). Briefly, mouse BM cells from an individual animal were seeded at 1 × 10^4^ cells/dish in 35 cm cell culture dishes (BD Biosciences). Plates were scored for total colony-forming units (CFUs) and colonies of erythroid burst-forming units (BFUs-E), granulocyte–macrophage (CFU-GM), and mixed-lineage (CFU-GEMM) after culturing for 8–10 days at 37°C in 5% CO_2_.

### Mouse serum cytokine assay

Serum samples were subjected to a mouse cytokine antibody array analysis, detecting mouse proteins semi-quantitatively using the mouse cytokine array C3 (RayBiotech, Inc., Peachtree Corners, GA) according to the manufacturer’s instructions. The kit provided antibodies for the detection of 62 cytokines, chemokines, growth factors, and soluble receptors of cytokines. In brief, the array membrane coated with cytokine antibodies was first blocked with a blocking buffer and then incubated with 1.2 ml of serum at 4°C overnight. After washing and incubation with biotin-conjugated secondary antibody for 2 h at room temperature, the membranes were washed again and incubated with horseradish peroxidase-conjugated streptavidin. The membranes were developed using an enhanced chemiluminescence solution, and the images were captured with the ChemiDoc MP Imaging System (Bio-Rad, Hercules, CA). A total of 62 cytokines/proteins, namely, Axl, BLC, CD30-ligand, CD30, CD40, CRG-2, CTACK, CXCL16, eotaxin-1, eotaxin-2, Fas-ligand, fractalkine, G-CSF, GM-CSF, IFNγ, IGFBP-3, IGFBP-5, IGFBP-6, IL-1α, IL-1β, IL-2, IL-3, IL-3Rβ, IL-4, IL-5, IL-6, IL-9, IL-10, IL-12p40/70, IL-12p70, IL-13, IL-17A, KC, leptin, leptin R, LIX, L-selectin, lymphotactin, MCP-1, MCP-5, M-CSF, MIG, MIP-1α, MIP-1γ, MIP-2, MIP-3α, MIP-3β, platelet factor-4, p-selectin, RANTES, SCF, SDF-1α, TNF-R1, TNF-RII, TARC, I-309, TECK, TIMP-1, TNFα, TPO, VCAM-1, and VEGF-A, were measured. After chemiluminescence detection, the signal intensity for each antigen-specific antibody spot was quantified using Image Lab Densitometry Software (version 6.0.1, Bio-Rad, Hercules, CA). Initially, the numerical densitometry value was subtracted to eliminate background signals, utilizing the negative and blank spots. Subsequently, the densitometric value of each target protein was normalized to the positive control spots on each membrane. The difference of each protein target between the MAAM and vehicle groups was calculated by dividing the normalized densitometric value of the MAAM group by that of the vehicle group [V_(MAAM)_/V_(Vehicle)_]. In addition, sera from the 9.5 Gy irradiated female mice were analyzed using the Mouse Cytokine/Chemokine 44-Plex Discovery Assay® Array (MD44) (Eve Technologies, Calgary, Alberta, Canada) as described (17).

### Statistical analysis

The 30-day survival of mice was analyzed using the Kaplan–Meier analysis. Fisher’s exact test was used to compare survival among groups at the end of 30 days after TBI. For CBC and cytokine data, differences between the means were compared using the analysis of variance (ANOVA) and Student’s *t*-tests. A *p*-value of <0.05 was considered statistically significant. Results were presented as means ± standard errors of the mean.

## Results

### MAAM administration increased survival in CD2F1 male mice after 9.0 Gy TBI

**Figure 1:**
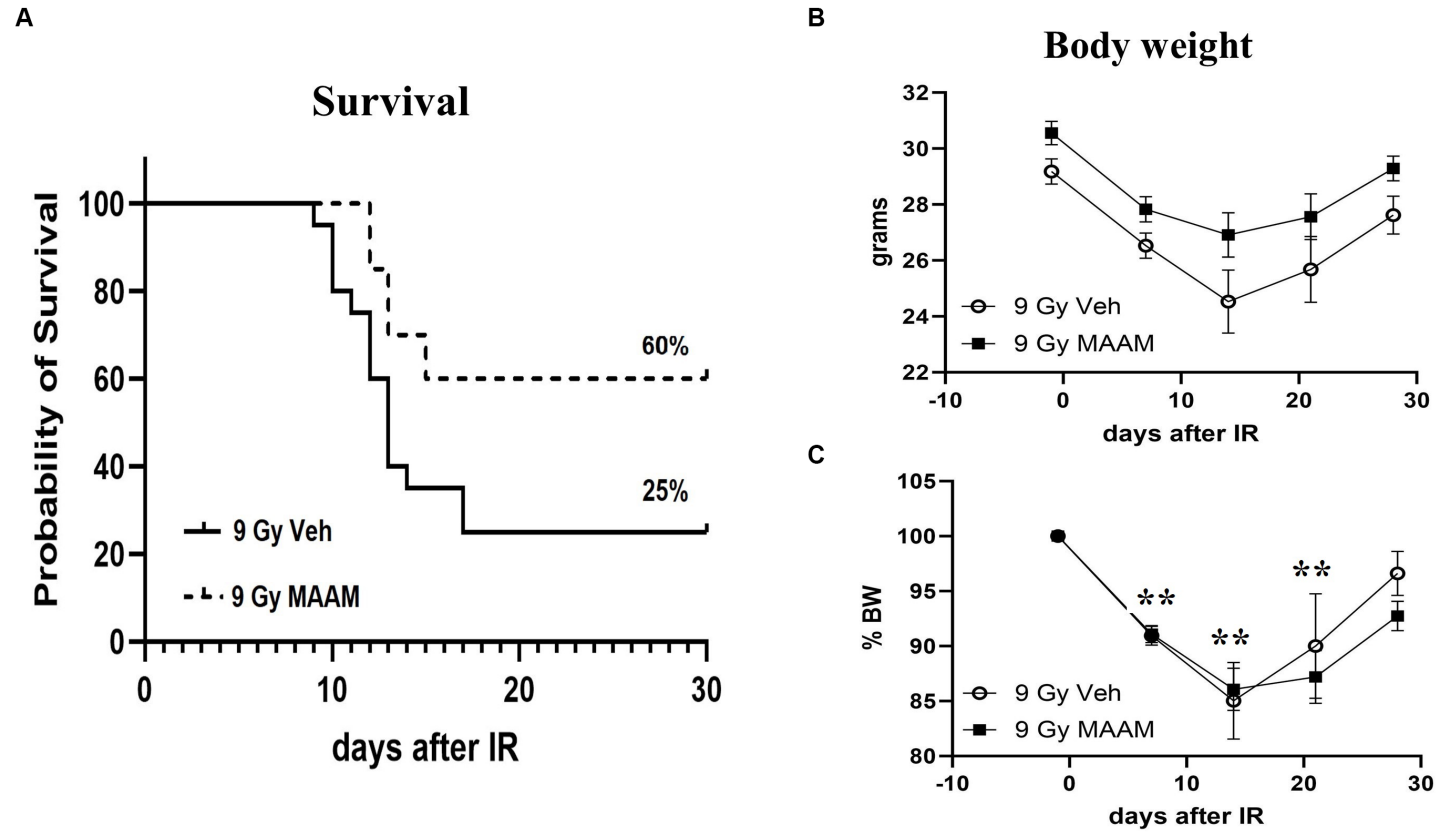
MAAM administration increased survival in male CD2F1 mice after total-body irradiation. The Kaplan–Meier survival curves show a 30-day survival rate in vehicle- or MAAM-treated male mice after 9.0 Gy TBI **(A)**. Treatment with MAAM significantly increased survival compared with vehicle treatment after 9.0 Gy TBI (*p* < 0.05). *N* = 20 mice for each group. **(B)** The body weight changed from day −1 to day 28 after exposure to 9.0 Gy of radiation and treated with vehicle control or MAAM, respectively. **(C)** Normalized body weight data is depicted as the percentage of the basal body weight (% BW). A significant reduction in body weight was observed following 9.0 Gy TBI. MAAM treatment had no significant effects compared to the vehicle control group at any time point. **p < 0.01. Means ± SEM.

CD2F1 male mice exposed to 9.0 Gy TBI were treated with MAAM or saline as vehicle control (200 μl/mouse) through oral gavage once daily for 14 days, starting at 24-h post-TBI. Animal survival was monitored for 30 days. The 30-day survival data are summarized in Figure 1A. The radiation exposure caused severe lethality in these mice and resulted in 75% animal death after 9.0 Gy in the vehicle control group. Treatment with MAAM after 9.0 Gy TBI significantly increased animal survival from 25% in the vehicle-treated group to 60% in the MAAM-treated group (*p* < 0.05).

Body weight loss is a critical indicator of radiation toxicity; therefore, it was measured from day −1 to day 28 after TBI. Figure 1B shows the body weight changing from day −1 to day 28 after exposure to 9.0 Gy radiation with vehicle or MAAM treatment, respectively. After normalization of body weight data, depicted as the percentage of body weight (% BW) compared to the basal body weight (day −1) in Figure 1C, it was evident that 9.0 Gy TBI significantly decreased body weight at days 7, 14, and 21 post-irradiation. However, administration of MAAM did not alleviate the body weight loss compared to the vehicle control group.

### MAAM treatment increased bone marrow hematopoietic stem and progenitor cells in CD2F1 male mice after 9.0 Gy TBI

**Figure 2:**
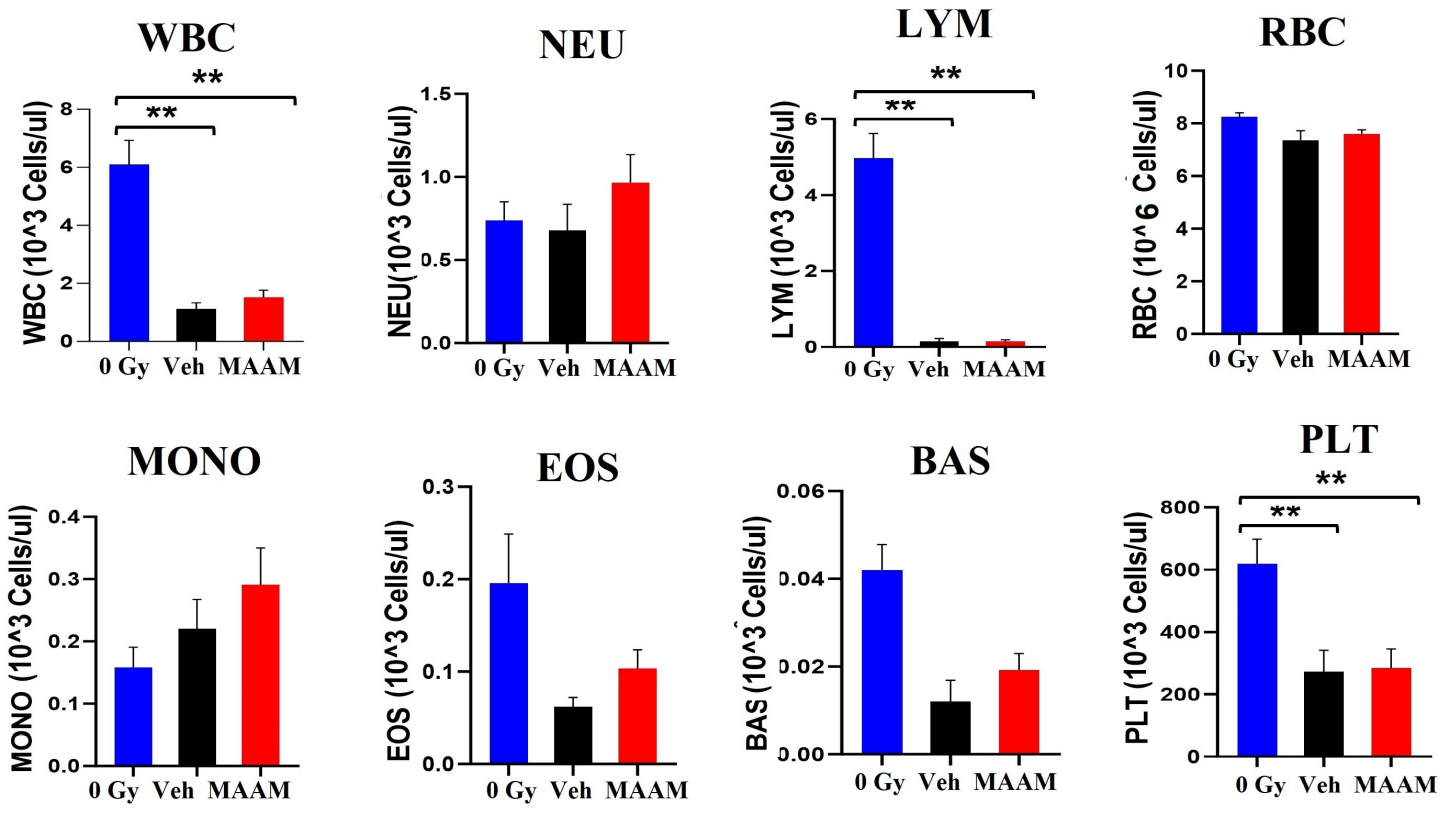
Complete blood cell counts (CBCs) including white blood cells (WBCs), neutrophils (NEU), lymphocytes (LYM), monocytes (MONO), eosinophils (EOS), basophils (BAS), red blood cells (RBCs), and platelets (PLTs) measured in 30-day surviving mice treated with MAAM or vehicle after 9.0 Gy TBI. WBC, LYM, and PLT are significantly lower than the non-irradiated male mouse control, and no significant differences in CBC between vehicle and MAAM-treated groups. **p < 0.01. Means ± SEM. *N* = 5–12/group.

Next, complete blood counts (CBCs) and BM clonogenicity from the surviving mice 30 days after 9.0 Gy TBI were evaluated. Figure 2 shows the total white blood cells (WBCs), neutrophils (NEU), lymphocytes (LYM), monocytes (MONO), eosinophils (EOS), basophils (BAS), red blood cells (RBCs), and platelets (PLTs) measured in the whole blood of mice that received MAAM or vehicle treatment. The counts of WBC, LYM, and PLT in the 30-day surviving mice after 9.0 Gy TBI remained significantly lower compared to the non-irradiated (0 Gy) control mice. Furthermore, the 95% intervals of the hematological parameters in CD2F1 male mice are shown in Table 2 (*N* = 5–12/group). There were no significant differences in CBC parameters between the surviving mice treated with MAAM and those treated with vehicle control.

**Table 2:** Hematological parameters in CD2F1 male mouse blood.

**CD2F1 male**		**WBC** (K/μL)	**NEU** (K/μL)	**LYM** (K/μL)	**MONO** (K/μL)	**EOS** (K/μL)	**BAS** (K/μL)	**RBC** (M/μL)	**PLT** (K/μL)
Reference values	Mean	6.10	0.74	4.97	0.16	0.20	0.04	8.25	618.80
95% interval	Low	4.47	0.52	3.67	0.09	0.09	0.03	7.93	462.78
	High	7.73	1.96	6.26	0.22	0.30	0.05	8.56	774.82
	*N*	5	5	5	5	5	5	5	5
9.0 Gy vehicle	Mean	1.12	0.68	0.15	0.22	0.06	0.01	7.36	271.80
95% interval	Low	0.71	37	0.005	0.12	0.042	0.002	6.66	210.48
	High	1.53	0.99	0.30	0.32	0.082	0.022	8.06	333.12
	N	5	5	5	5	5	5	5	5
9.0 Gy MAAM	Mean	1.53	0.97	0.15	0.29	0.10	0.02	7.59	284.60
95% interval	Low	1.06	0.64	0.06	0.17	0.06	0.002	7.28	164.43
	High	1.99	1.29	0.23	0.41	0.14	0.036	7.90	404.73
	N	12	12	12	12	12	12	12	12

Complete blood cell counts (CBCs) including white blood cells (WBCs), neutrophils (NEU), lymphocytes (LYM), monocytes (MONO), eosinophils (EOS), basophils (BAS), red blood cells (RBCs), and platelets (PLTs) measured in 30-day surviving mice treated with MAAM or vehicle after 9.0 Gy TBI. Reference values from non-irradiated (0 Gy) animal samples.

**Figure 3:**
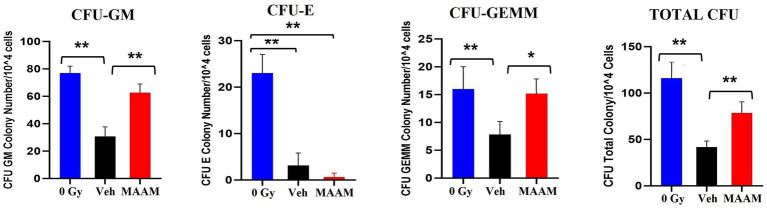
Clonogenicity assay of the vehicle- or MAAM-treated male mice after 9.0 Gy TBI. Colony-forming assay was performed with 1 × 10^4^ BM cells/each animal, and clonogenicity was scored and compared among samples collected from 0 Gy male mice, vehicle-treated, and MAAM-treated male mice after 9.0 Gy TBI. MAAM significantly increased total colony numbers in 30-day surviving animals with high CFU-GM and the early-stage progenitors CFU-GEMM colonies. *p < 0.05; **p < 0.01. Means ± SEM. *N* = 5/group.

We further compared the effects of MAAM treatment on the survival of BM hematopoietic progenitor cells in these mice. BM cells were collected from the femurs and humeri of 30-day surviving mice treated with MAAM or vehicle control. Total live BM myeloid cells in the pooled samples from each mouse were quantified with Trypan blue staining. Colony assay was performed with 1 × 10^4^ BM cells/animal and clonogenicity was scored and compared among samples as shown in Figure 3. MAAM treatment significantly increased the total colony number in 30-day surviving animals with high CFU-GM and early-stage progenitors CFU-GEMM colonies compared to the vehicle control group. This increase reached similar levels of CFU-GM and CFU-GEMM as observed in non-irradiated (0 Gy) mice. However, CFU-E colonies were not recovered from the MAAM-treated group 30 days after 9.0 Gy TBI (*N* = 5/group).

### MAAM regulated serum cytokine levels in CD2F1 male mice after 9.0 Gy TBI

**Figure 4:**
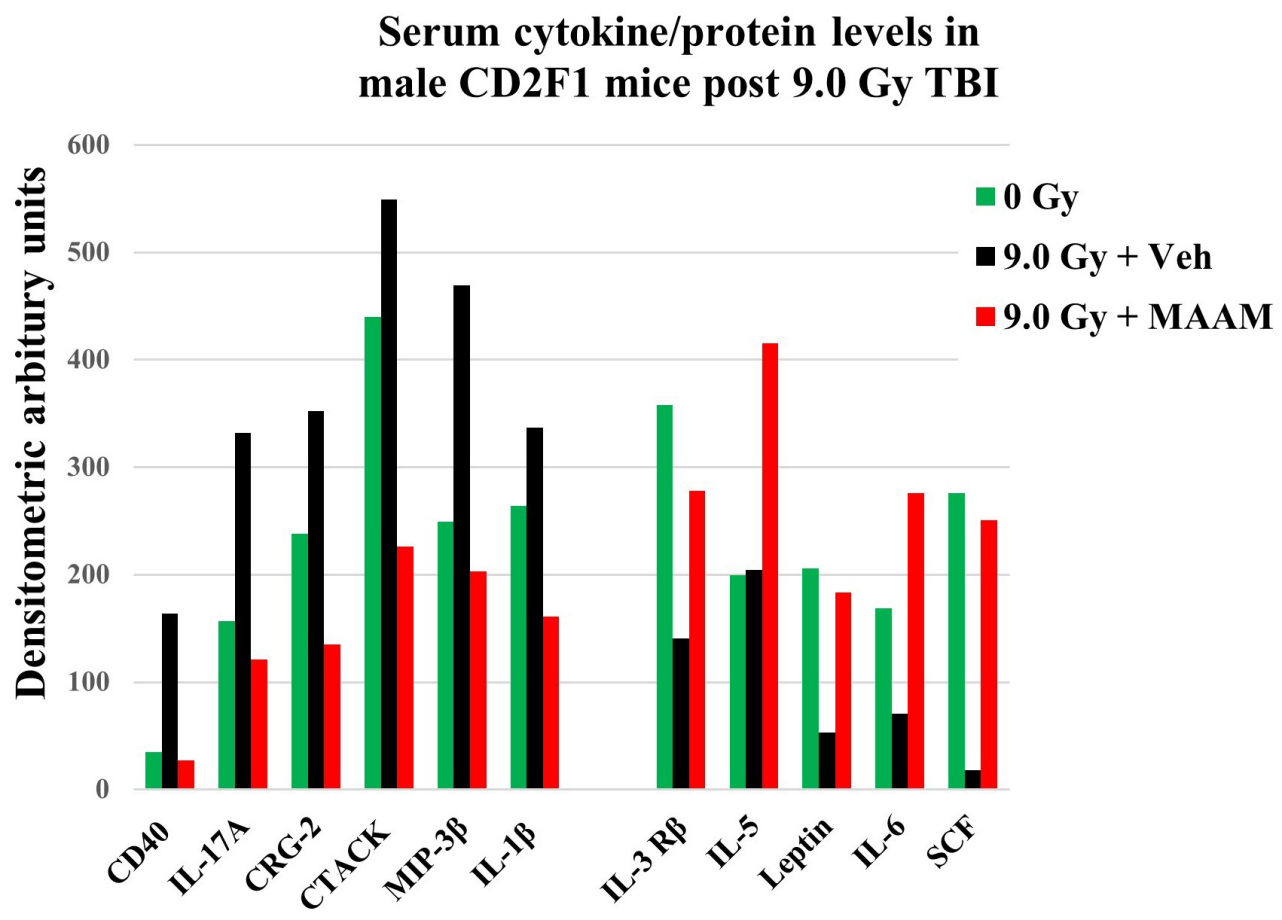
MAAM regulated serum cytokine/protein levels in the CD2F1 male mice after 9.0 Gy TBI. A cytokine antibody array with a total of 62 cytokines/ proteins was used to measure the pooled sera (5 mice/group) from 30-day survival mice treated with vehicle or MAAM after 9.0 Gy TBI. Serum cytokine/protein levels from 0 Gy non-irradiated mice served as a normal healthy control. Each protein level was represented by its normalized densitometric values in arbitrary units. A total of 11 out of 62 serum cytokines/proteins were changed by MAAM in the irradiated mice with [V_(MAAM)_/V_(Vehicle)_] < 0.5 or ≥ 2. Treatment with MAAM decreased six and increased five cytokines/proteins after 9.0 Gy TBI compared with the vehicle-treated group.

Radiation exposure induces cytokine cascade activation to modulate the immune system (14, 18). Next, we investigated whether MAAM modulated cytokine levels in the surviving male mice exposed to the lethal dose of 9.0 Gy TBI using the cytokine antibody array. Mouse serum was collected on day 30 after irradiation with MAAM or vehicle control treatment. Because the C3 cytokine array required 1.2 ml of serum per assay, and each mouse could only produce a limited amount, the levels of cytokines/proteins were determined from the pooled sera of five individual mice per group. The results in Table 3 and Figure 4 demonstrated that, in comparison with the vehicle control group, 11 out of the 62 cytokine/protein levels were changed by the MAAM treatment [V_(MAAM)_/V_(Vehicle)_ < 0.5 or ≥ 2]. MAAM treatment decreased six cytokines/proteins, namely, CD40, IL-17A, CRG-2, CTACK, MIP-3β, and IL-1β and increased five cytokines/proteins, namely, IL-3 Rβ, IL-5, leptin, IL-6, and SCF. Among the increased cytokines, SCF increased 13.8-fold in the MAAM-treated versus the vehicle-treated group. Treatment with MAAM maintained the majority of the 11 cytokine/ protein levels affected by 9.0 Gy TBI (either decreased or increased) comparable to those in non-irradiated (0 Gy) healthy mouse serum (Figure 4).

**Table 3:** Cytokine/protein levels changed by MAAM treatment in the male mouse serum after 9.0 Gy TBI.

	**Reference values (0 Gy)**	**Vehicle (9.0 Gy)**	**MAAM (9.0 Gy)**	**V**_**(MAAM)**_ **/V**_**(Vehicle)**_ **(9.0 Gy)**
**CD40**	34.9	163.9	27.1	0.17
**IL-17A**	156.7	332.3	121.2	0.36
**CRG-2**	268.3	352.3	135.2	0.38
**CTACK**	440.0	549.1	226.4	0.41
**MIP-3β**	249.4	469.3	203.1	0.43
**IL-1β**	263.8	337.2	160.9	0.48
**IL-3 Rβ**	357.6	141.0	277.9	1.97
**IL-5**	199.5	204.5	415.3	2.03
**Leptin**	205.7	52.8	183.4	3.47
**IL-6**	168.6	70.7	275.8	3.90
**SCF**	275.6	18.2	250.8	13.82

A cytokine antibody array with a total of 62 cytokines/proteins was used to test the pooled serum from 30-day survival male mice treated with vehicle or MAAM after TBI. Reference values were from non-irradiated (0 Gy) animal samples. Cytokines/proteins with [V_(MAAM)_/V_(Vehicle)_] < 0.5 or ≥2 are shown here. The normalized densitometric value of each target is shown.

### MAAM administration did not increase survival or change body weight loss in CD2F1 female mice after TBI

**Figure 5:**
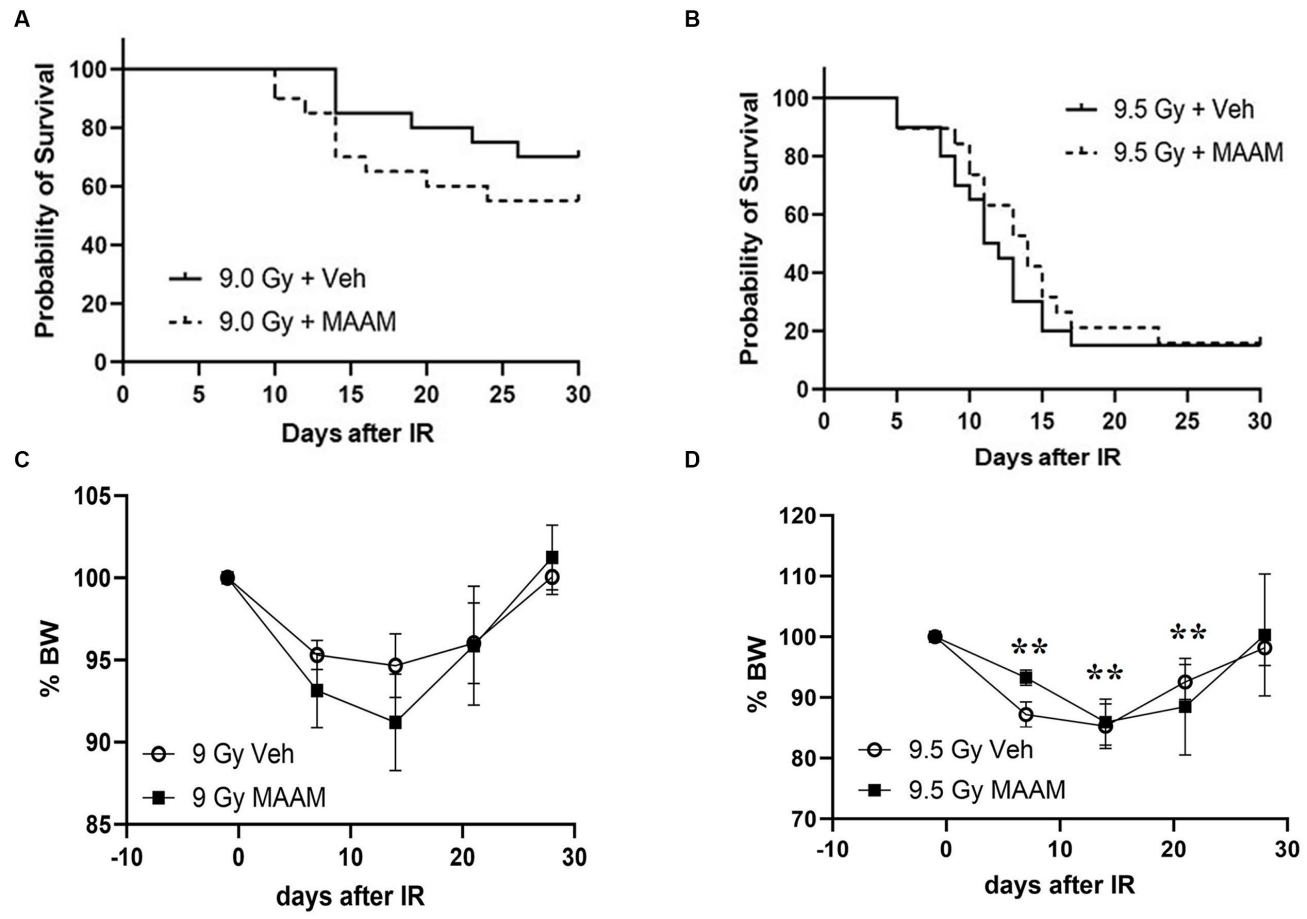
MAAM administration did not increase the survival of female CD2F1 mice after total-body irradiation. The Kaplan–Meier survival curves show 30-day survival rates in vehicle- or MAAM-treated female mice after 9.0 Gy **(A)** and 9.5 Gy **(B)** TBI (*N* = 20). Normalized body weight data depicted as the percentage of basal body weight (% BW) are shown in **(C,D)**. A significant reduction in body weight was observed following 9.5 Gy TBI **(D)**. Treatment with MAAM did not affect the body weight loss observed at all time points after 9.0 Gy or 9.5 Gy TBI. **p < 0.01. Means ± SEM.

We next compared the alleviating effects of MAAM on survival and body weight loss in CD2F1 female mice after 9.0 Gy TBI. CD2F1 female mice at age 12–14 weeks were subjected to TBI. MAAM or vehicle treatment was started at 24-h post-TBI, once daily for 14 days. Survival was tracked for 30 days, and body weight measurements were performed from day −1 to day 28 after TBI. Figure 5A shows that 9.0 Gy TBI resulted in 75% survival in the female mice treated with the vehicle at day 30, indicating more radiation resistance in female mice than male mice. Treatment with MAAM did not show improvement in survival in the irradiated female mice compared to those treated with the vehicle. To further investigate the effects of MAAM on the survival of female mice, 9.5 Gy TBI was used in a subsequent experiment. TBI at 9.5 Gy decreased female mice survival rate from 75% (after 9.0 Gy) to 15% (after 9.5 Gy) in the vehicle control group on day 30, as shown in Figure 5B. However, the alleviating effects of MAAM were not observed in these mice. Furthermore, normalized body weight data depicted as the percentage of body weight (% BW) compared to the basal body weight (day −1) are shown in Figures 5C,D. A significant reduction in body weight was observed on days 7, 14, and 21 following 9.5 Gy TBI (Figure 5D). However, treatment with MAAM did not alleviate the body weight loss compared to the vehicle-treated group at all time points after 9.0 Gy or 9.5 Gy TBI.

**Figure 6:**
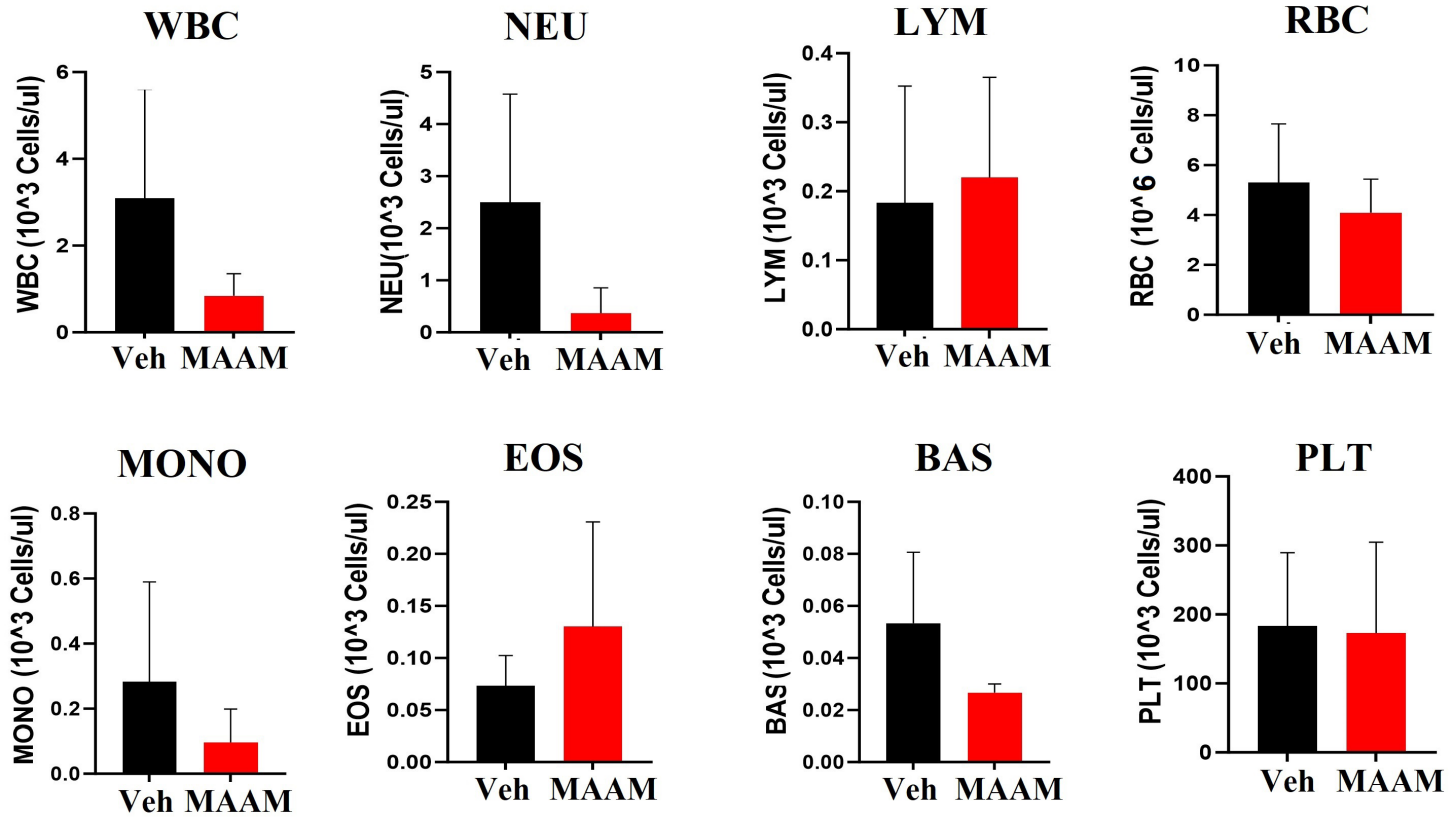
Complete blood cell counts (CBCs) including white blood cells (WBCs), neutrophils (NEU), lymphocytes (LYM), monocytes (MONO), eosinophils (EOS), basophils (BAS), red blood cells (RBCs), and platelets (PLTs) were measured in 30-day surviving female mice treated with MAAM or vehicle after 9.5 Gy TBI. No significant differences in CBC between groups were observed.

The CBC was also performed on the day 30 surviving female mice after 9.5 Gy TBI. No significant changes in CBC parameters were observed in the surviving mice treated with vehicle control or MAAM after 9.5 Gy TBI (Figure 6 and Table 4).

**Table 4:** Hematological parameters in CD2F1 female mouse blood.

**CD2F1 female**		**WBC** (K/μL)	**NEU** (K/μL)	**LYM** (K/μL)	**MONO** (K/μL)	**EOS** (K/μL)	**BAS** (K/μL)	**RBC** (M/μL)	**PLT** (K/μL)
Reference values*	Mean	8.72	1.63	6.52	0.39	0.19	NA	10.91	NA
95% interval	Low	8.33	1.51	6.23	0.34	0.16		10.85	
	High	9.11	1.74	6.82	0.44	0.21		10.97	
	*N*	275	275	275	275	275		284	
9.5 Gy vehicle	Mean	3.09	2.50	0.18	0.28	0.07	0.05	5.30	183.30
95% interval	Low	0.25	0.146	0.00	0.00	0.67	0.00	0.69	0.00
	High	5.93	4.85	0.37	0.63	0.79	0.11	9.91	391.13
	*N*	3	3	3	3	3	3	3	3
9.5 Gy MAAM	Mean	0.84	0.37	0.22	0.10	0.13	0.03	4.09	172.70
95% interval	Low	0.26	0.00	0.06	0.00	0.00	0.01	1.47	0.00
	High	1.42	0.92	0.38	0.21	0.33	0.08	6.72	431.70
	*N*	3	3	3	3	3	3	3	3

Complete blood cell counts (CBCs) including white blood cells (WBCs), neutrophils (NEU), lymphocytes (LYM), monocytes (MONO), eosinophils (EOS), basophils (BAS), red blood cells (RBCs), and platelets (PLTs) measured in 30-day CD2F1 female surviving mice treated with MAAM or vehicle after 9.5 Gy TBI. *Reference values: 0 Gy female CD2F1 hematology data were derived from Swoyer et al. (19).

### Cytokine level changes in CD2F1 female mouse serum after 9.5 Gy TBI with MAAM or vehicle treatment

**Figure 7:**
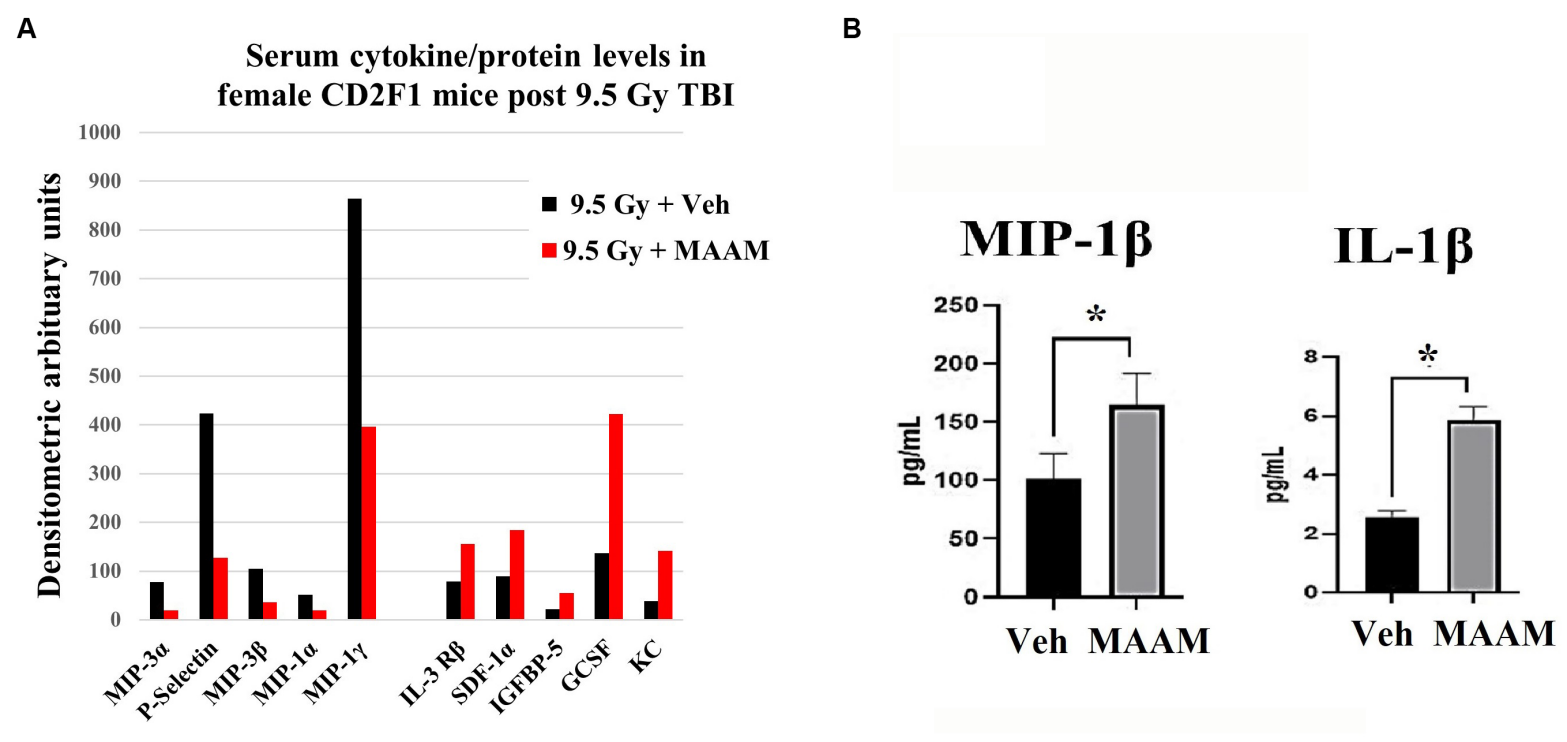
MAAM regulated serum cytokine levels in female mice after 9.5 Gy TBI. **(A)** A cytokine antibody array with a total of 62 cytokines/ proteins was used to test the pooled sera (3 mice/group) from 30-day surviving female mice treated with vehicle or MAAM after TBI. Each protein level was represented by its normalized densitometric values in arbitrary units. A total of 10 out of 62 cytokines/proteins were changed by MAAM with [V_(MAAM)_/V_(Vehicle)_] < 0.5 or ≥2. Treatment with MAAM decreased five and increased five cytokines compared with the vehicle-treated group after 9.5 Gy TBI. **(B)** MAAM significantly increased MIP-1β and IL-1β levels in the serum of female mice after 9.5 Gy TBI. *p < 0.05. Means ± SEM. *N* = 3/group.

Finally, mouse serum cytokines/proteins were measured in the 30-day surviving female mice after 9.5 Gy TBI using the cytokine antibody array. Levels of cytokines/proteins were determined from the pooled sera of three surviving mice per group. Interestingly, the cytokine/protein profiles in the male and female mice in response to MAAM and vehicle control treatment after radiation exposure were very different. The results in Table 5 and Figure 7A demonstrated that, in comparison with the vehicle control group, 10 out of the 62 cytokine/protein levels were changed by MAAM treatment [V_(MAAM)_/V_(Vehicle)_ < 0.5 or ≥2]. Treatment with MAAM after 9.5 Gy TBI decreased five cytokines/proteins, namely, MIP3α, P-selectin, MIP3β, MIP-1α, and MIP-1γ, and increased five cytokines /proteins, namely, IL-3 Rβ, SDF-1α, IGFBP-5, G-CSF, and KC. In addition, the serum cytokine/protein levels after 9.5 Gy irradiation with MAAM or vehicle treatment were measured using the Mouse Cytokine/Chemokine 44-Plex Discovery Assay® Array (MD44) (Eve Technologies, Calgary, Alberta, Canada) with *N* = 3 mice per group. Treatment with MAAM significantly increased the levels of MIP-1β and IL-1β compared to the vehicle control group in female mice, as shown in Figure 7B.

**Table 5:** Cytokine levels changed by MAAM treatment in female mouse serum after 9.5 Gy TBI.

	**Vehicle (9.5 Gy)**	**MAAM (9.5 Gy)**	**V**_**(MAAM)**_ **/V**_**(Vehicle)**_ **(9.5 Gy)**
**MIP-3α**	77.8	19.7	0.25
**P-Selectin**	423.8	126.9	0.30
**MIP-3β**	105.3	36.3	0.34
**MIP-1α**	51.3	19.7	0.38
**MIP-1γ**	864.3	396.3	0.46
**IL-3 Rβ**	78.6	155.6	1.98
**SDF-1α**	89.9	184.2	2.05
**IGFBP-5**	22.3	55.6	2.50
**G-CSF**	137.4	422.0	3.07
**KC**	38.4	141.5	3.69

A cytokine antibody array with a total of 62 cytokines/proteins was used to test the pooled serum from 30-day survival female mice treated with vehicle or MAAM after 9.5 Gy TBI. Cytokines/proteins with [V_(MAAM)_/V_(Vehicle)_] < 0.5 or ≥2 are shown here. The normalized densitometric value of each target is shown.

### The different effects of MAAM in CD2F1 male and female mice after lethal doses of TBI

Finally, we summarized the distinct effects of MAAM in male and female CD2F1 mice following radiation exposure. Table 6 presents a comparison of mortality rates, while Table 7 outlines the divergent cytokine/protein profiles between the male and female mice treated with MAAM versus the vehicle control after irradiation. In Table 6, it was observed that female mice exhibited greater radiation resistance compared to male mice, as shown in the vehicle-treated groups. For instance, following radiation at 9.0 Gy TBI, the male mice had a mortality rate of 75%, whereas only 25% of the female mice succumbed. Elevating the radiation dose from 9.0 Gy to 9.5 Gy increased the female mice’s mortality from 25 to 85%. Treatment with MAAM significantly augmented the survival of male mice, decreasing the mortality from 75 to 40%, whereas no survival benefit was observed in the female mice.

**Table 6:** Effects of MAAM on 30-day survival of CD2F1 mice of different sexes.

**Mortality rate**	**Male CD2F1 9.0 Gy**	**Female CD2F1 9.0 Gy**	**Female CD2F1 9.5 Gy**
**Vehicle-treated**	75%	25%	85%
**MAAM-treated**	40%	35%	85%
***p*-value**	*p* < 0.05	*p* > 0.05	NA

Male and female mice receiving ^60^Co-γ total body irradiation (TBI) were treated with MAAM or saline as a vehicle control once daily for 14 days starting at 24-h post-TBI. Animal survival was monitored for 30 days and compared. *N* = 20/group of mice.

**Table 7:** Divergent cytokine/protein profiles between male and female mice treated with MAAM versus the vehicle control after a lethal dose of TBI.

**Cytokine/protein** [V_(MAAM)_/V_(Vehicle)_]	**Male CD2F1 mice 9.0 Gy**	**Female CD2F1 mice 9.5 Gy**
**Decreased cytokines/proteins**	CD40 (0.17)	MIP-3α (0.25)
	IL-17A (0.36)	P-Selectin (0.30)
	CRG-2 (0.38)	MIP-3β (0.34)
	CTACK (0.41)	MIP-1α (0.38)
	MIP-3β (0.43)	MIP-1γ (0.46)
	IL-1β (0.48)	
**Increased cytokines/proteins**	IL-3 Rβ (1.97)	IL-3 Rβ (1.98)
	IL-5 (2.03)	SDF-1α (2.05)
	Leptin (3.47)	IGFBP-5 (2.50)
	IL-6 (23.90)	G-CSF (3.07)
	SCF (13.8)	KC (3.69)

A cytokine antibody array with a total of 62 cytokines/proteins was used to test the pooled serum from 30-day survival male and female mice treated with vehicle or MAAM after TBI. Cytokines/proteins with [V_(MAAM)_/V_(Vehicle)_] < 0.5 or ≥ 2 are shown here. The ratio of [V_(MAAM)_/V_(Vehicle)_] of each target is shown in the parenthesis.

In Table 7, the cytokine/ protein profiles in male and female mice in response to MAAM treatment after radiation exposure were very different. Among the 10 or 11 proteins changed by MAAM treatment in both sexes, only 2 were identical (with MIP-3β decreased in both sexes and IL-3 Rβ increased in both sexes). The remaining eight or nine proteins were different between the sexes. Particularly, MAAM elevated SCF levels by 13.8-fold and leptin levels by 3.5-fold in the male mice, whereas no clear changes were observed for SCF and leptin in the female mice.

## Discussion

The risks associated with radiation-induced injuries are multifaceted and can lead to various health issues. These risks depend on factors such as the type, dose, and dose rate of radiation, as well as an individual’s variability in biological responses to radiation exposure, which includes differences in sex, age, immune response, and metabolism. Consequently, there is an urgent demand for new MCMs capable of effectively mitigating and treating multiple organ injuries induced by radiation in diverse populations following a radiological or nuclear mass casualty incident (10). Recently, Gupta et al. (3, 4) reported that treatment with AA-ORS could decrease radiation-induced GI injury, increase electrolyte absorption, prevent antigen translocation, and reduce inflammation, subsequently improving intestinal mucosal barrier function in male NIH Swiss mice exposed to TBI.

Amino acids are fundamental components present in cells, acting as the basic elements of mammalian cellular machinery and serving as the building blocks for protein and enzyme synthesis. While mammals can synthesize 11 non-essential amino acids from metabolic intermediates, they lack the ability to produce 9 amino acids, thus requiring their acquisition of nutrients. These are known as essential amino acids (20–22). We have reported that specific amino acid permeases have been identified as playing crucial roles in determining the radiation sensitivity of *Cryptococcus neoformans* (2). Utilizing a combination of amino acids as a mitigating agent through oral administration for acute radiation-induced injuries in casualties could represent a novel strategy. If these amino acids demonstrate the ability to mitigate multiple organ injuries, it could pave the way for an innovative approach to radiation injury treatment. Therefore, we conducted the tests to assess the alleviating effects of MAAM on the survival of CD2F1 male and female mice following exposure to lethal doses of TBI. This MAAM includes five amino acids (threonine, valine, serine, tyrosine, and tryptophan) and an electrolyte solution indicated in Table 1. Our data showed that treatment with MAAM significantly increased the survival rate from 25% in the vehicle group to 60% after 9.0 Gy TBI in the CD2F1 male mice. Furthermore, MAAM treatment remarkably promoted the survival of BM hematopoietic stem and progenitor cells, as shown by significantly higher clonogenicity numbers in the MAAM-treated group than in the vehicle control-treated group. Our data and literature indicate that the alleviating effects of MAAM in mice are mediated not only through the GI system but also through the hematopoietic system. To further understand the mechanisms of MAAM in improving the survival of the male mice, we compared the levels of cytokines/proteins in the mouse serum from MAAM-treated and vehicle-treated 30-day surviving mice after 9.0 Gy TBI using a cytokine antibody array method. Data in Table 3 and Figure 4 demonstrate that MAAM treatment decreased six cytokines/proteins and increased five cytokines/proteins compared with the vehicle-treated group. MAAM treatment restored those cytokine/protein levels affected by 9.0 Gy TBI to unirradiated levels. Notably, SCF was among the five increased cytokines, showing a remarkable 13.8-fold increase following MAAM treatment. SCF is an essential hematopoietic cytokine that interacts with other cytokines to preserve the viability of hematopoietic stem and progenitor cells, influencing their entry into the cell cycle, proliferation, and differentiation. The elevation of SCF in serum may explain the mechanism of MAAM in alleviating radiation-induced injury. In addition, leptin levels were also increased by MAAM treatment in these mouse sera. Leptin, a hormone produced in adipocytes, plays a crucial role in maintaining a normal body weight and regulating energy expenditure (23–25). Our current data suggest that, in addition to the previously reported mitigative effects of GI-ARS in male Swiss mice, MAAM may also enhance the survival of male CD2F1 mice following radiation exposure by protecting against radiation-induced hematopoietic system failure. Moreover, findings from these two research groups, using distinct mouse strains and radiation sources, collectively demonstrate the radiation mitigative potential of MAAM in male mice post-radiation injury.

We subsequently assessed the effects of MAAM on the survival rates of CD2F1 female mice following 9.0 and 9.5 Gy TBI. Considering the recognized sex-based variations in normal tissue response and tolerance to radiation exposure (6, 8, 26), these differences could significantly influence the efficacy of identical countermeasures. TBI at 9.0 Gy led to a 25% mortality rate in the female CD2F1 mice treated with the vehicle control, whereas it caused a 75% mortality rate in the male CD2F1 mice, as depicted in Table 6. These findings suggest greater radiation resistance in the female mice compared to the male mice. However, a 25% lethality rate complicated the demonstration of a countermeasure’s efficacy in improving survival. It is well-known that even a slight increase in radiation dose can significantly elevate animal mortality. TBI at 9.5 Gy raised female mice’ mortality from 25% (at 9.0 Gy TBI) to 85%, which is a more ideal lethality rate to test the effects of MAAM in female mice. However, MAAM did not lead to improved survival in the CD2F1 female mice after 9.0 or 9.5 Gy TBI, suggesting that there is a sex-dependent effect of MAAM as a radiation mitigator. Moreover, the analysis of mouse serum cytokine levels in female mice revealed distinct cytokine expression profiles compared to male mice at 30 days after radiation. The level of G-CSF was increased, but SCF was not changed by MAAM in the irradiated female mice. Radiation-induced cytokine/chemokine expression in mouse serum has been used as a biomarker to predict radiation-induced tissue injuries. However, the effects of sex on cytokine/chemokine responses after radiation exposure remain poorly defined. Whether the different cytokine expressions in different sexes may impact the prognosis of mice requires further investigation (7).

The sex differences in response to radiation exposure have been brought to attention, but the underlying factors that contribute to these differences are still not fully understood. Recently, Taliaferro et al. (27) summarized a report from “The Sex Differences in Radiation Research workshop” and emphasized that “investigators in radiation and radiation countermeasure research field should accommodate both sexes in all stages of research to ensure that the outcome is robust, reproducible, and accurate, and will benefit public health.” Sex differences in the utilization of amino acids vary across species, with distinct strategies observed in moths, mice, rats, and humans for the allocation, oxidation, and metabolism of dietary amino acids (26, 28–30). Data from our study demonstrated the significant differences in the alleviating effects of MAAM on the survival of different sexes of mice, suggesting that the different sexes of animals may require distinct mitigative amino acid combination treatments based on their different metabolism, redox balance, and epigenetic regulation. Understanding how these differences may impact radiation countermeasure function is an important step toward future MCM development.

## Conclusion

Oral administration of MAAM significantly increased survival following a lethal dose of TBI in male CD2F1 mice. MAAM treatment exhibited a protective effect against radiation-induced BM hematopoietic cell injury and elevated levels of SCF and leptin in the male mice, potentially contributing to improved animal survival. However, the observed alleviating effects of MAAM were not apparent in female CD2F1 mice exposed to lethal doses of radiation. These findings highlight the potential necessity for tailored mitigative amino acid combination treatments based on the sex of the animals. Further investigation into the mechanisms underlying MAAM’s radiation mitigative effects is currently underway in our research group. These discoveries hold promise for pioneering strategies in the development of mitigative drugs, offering benefits to both male and female victims of radiation injury.

## Data availability statement

The raw data supporting the conclusions of this article will be made available by the authors, without undue reservation.

## Ethics statement

The animal study was approved by The Uniformed Services University of the Health Sciences (USUHS)-Institutional Animal Care and Use Committee (IACUC). The study was conducted in accordance with the local legislation and institutional requirements.

## Author contributions

MX: Conceptualization, Data curation, Formal analysis, Funding acquisition, Investigation, Methodology, Supervision, Validation, Writing – original draft, Writing – review & editing. LH: Data curation, Formal analysis, Investigation, Methodology, Writing – review & editing. AZ: Data curation, Investigation, Methodology, Validation, Writing – review & editing. BL: Data curation, Formal analysis, Investigation, Methodology, Validation, Writing – review & editing. MZ: Data curation, Formal analysis, Methodology, Validation, Writing – review & editing. LW: Data curation, Investigation, Methodology, Validation, Writing – review & editing. WC: Data curation, Formal analysis, Investigation, Methodology, Supervision, Validation, Writing – original draft, Writing – review & editing.
